# Degradation of Polysaccharides from *Grateloupia filicina* and Their Antiviral Activity to Avian Leucosis Virus Subgroup J

**DOI:** 10.3390/md15110345

**Published:** 2017-11-03

**Authors:** Yuhao Sun, Xiaolin Chen, Ziqiang Cheng, Song Liu, Huahua Yu, Xueqin Wang, Pengcheng Li

**Affiliations:** 1 Key Laboratory of Experimental Marine Biology, Institute of Oceanology, Chinese Academy of Sciences, No. 7 Nanhai Road, Qingdao 266071, China; 18669884128@163.com (Y.S.); sliu@qdio.ac.cn (S.L.); yuhuahua@qdio.ac.cn (H.Y.); xueqinwang@qdio.ac.cn (X.W.); 2 University of Chinese Academy of Sciences, Beijing 100049, China; 3 Laboratory for Marine Drugs and Bioproducts of Qingdao National Laboratory for Marine Science and Technology, No. 7 Nanhai Road, Qingdao 266071, China; 4 College of Animal Science and Veterinary Medicine, Shandong Agricultural University, No. 61 Daizong Road, Taian 271018, China; czqsd@126.com

**Keywords:** *Grateloupia filicina*, degradation, low molecular weight polysaccharides, antiviral activity, ALV-J

## Abstract

In this study, polysaccharides from *Grateloupia filicinia* (GFP) were extracted and several low molecular weight (Mw) *G. filicina* polysaccharides (LGFPs) were prepared by the hydrogen peroxide (H_2_O_2_) oxidation method. Additionally, the effect of different experimental conditions on the degradation of GFP was determined. Results showed that the GFP degradation rate was positively related to H_2_O_2_ concentration and temperature, and negatively related to pH. Chemical analysis and Fourier transform infrared spectra (FT-IR) of GFP and LGFPs showed that the degradation caused a slight decrease of total sugar and sulfate content. However, there was no obvious change for monosaccharide contents. Then, the anti-ALV-J activity of GFP and LGFPs were determined in vitro. Results revealed that all of the samples could significantly inhibit ALV-J and lower Mw LGFPs exhibited a stronger suppression, and that the fraction LGFP-3 with Mw 8.7 kDa had the best effect. In addition, the reaction phase assays showed that the inhibition effect was mainly because of the blocking virus adsorption to host cells. Moreover, real-time PCR, western-blot, and IFA were further applied to evaluate the blocking effects of LGFP-3. Results showed that the gene relative expression and gp85 protein for LGFPS-3 groups were all reduced. Data from IFA showed that there was less virus infected cells for 1000 and 200 μg/mL LGFPS-3 groups when compared to virus control. Therefore, lower Mw polysaccharides from *G. filicina* might supply a good choice for ALV-J prevention and treatment.

## 1. Introduction

Avian leucosis virus subgroup J (ALV-J) is an immunosuppressive virus, which generally causes a diversity of tumors, such as hemangioma and myeloid leucosis in birds [1]. It has spread worldwide since the first time it was discovered in British broiler breeds in 1988. In China, ALV-J was first detected and officially recorded in 1999 [2], and after that the ALV-J infection appeared around the country. The virus could infect chickens through both horizontal and vertical transmission; initially it was mainly found in turkeys and broilers, but recently it also brought disease and death in egg layers and broiler breeders [3], which caused heavy losses to the poultry breeding industry. Due to the complex genetic sequence and the antigenic variability, there are still no commercial vaccines against ALV-J infection [4,5,6]. Currently, the only way to prevent ALV-J is by quarantining and eliminating positive chickens. Therefore, it is urgent and necessary to find effective antiviral medicine to control ALV-J spread.

On the other hand, the red seaweed genus *Grateloupia*, which belongs to Halymeniaceae, is an intertidal alga [7], which is widely distributed in the countries of the west Pacific bank such as China, Japan, and South Korea. It has enormous commercial value because it may be used as raw materials for carrageenan. In addition, the protein content of *Grateloupia turuturu* can account for 20% of the dry weight therefore being of potential in the food industry [8]. Polysaccharide is another important chemical compound of *Grateloupia*. It has attracted wide attention in recent years due to its multiple biological activities. For example, Nikapitiya et al. reported a good anticoagulant activity of a sulfated polysaccharide that is extracted from *Grateloupia filicina* [9]. In addition, Yu et al. isolated an agaran-type polysaccharide from *G. filicina* and demonstrated that the polysaccharide carried antiangiogenic effects [10]. Further, the polysaccharides that are extracted from *G. livida* were reported to have antioxidant, antibacterial, and antisachistosomal activity [11,12]. Scientists also studied the antiviral activity of *Grateloupia* and found that polysaccharides obtained from *G. indica*, *G. filicina*, and *G. longilia* had anti-HSV-1, HSV-2, HIV-1, and dengue virus activity [13,14]. However, the studies on antiviral activity of the polysaccharide from *Grateloupia* were mainly focused on human suffering virus, and few studies aimed at finding the effects against poultry viruses.

Even though the high Mw *Grateloupia* polysaccharide has various biological activities, its huge Mw causes low solubility and tough organism absorption, which greatly restricts its application. Commonly, lower Mw algal polysaccharides are obtained by the degradation of high Mw polysaccharides. When compared with original high Mw polysaccharide, low Mw polymers are characterized by having a higher water solubility and stability, and easy organism absorption [15]. In order to obtain lower Mw polysaccharide a chain of polysaccharides is usually broken, as according to the 1 → 3, 1 → 4 and 1 → 6 glycosidic linkages fractures, and some of their original activity might be improved in different degrees [16,17,18]. Additionally, in recent years, it has been found that low Mw polysaccharides from seaweeds presented some effects on the suppression H1N1 influenza virus and HIV [19,20,21,22]. However, research on the anti-ALV-J activity of algal polysaccharides still scarce. Therefore, it would be innovative and promising applying the low Mw polysaccharide from *Grateloupia* to prevent ALV-J.

In this study, polysaccharides from *G. filicina* (GFP) were extracted by hot water extraction and were degraded via H_2_O_2_ oxidative degradation. The effect of different conditions (including concentration of H_2_O_2_, pH and temperature) on the degradation of GFP was determined. Several low Mw fractions of GFP (LGFPs) were prepared and their chemical composition was tested. Finally, the anti-ALV-J activity of LGFPs was explored. The results of this study would provide a possible useful application of seaweeds polysaccharides as a veterinary medicine in the prevention of ALV-J.

## 2. Results

### 2.1. Influence of Degradation Conditions on the Mws of Polysaccharides

The yield of the GFP that was extracted by traditional hot water extraction and alcohol precipitation methods was 34.44%. Freeze-dried GFP was dissolved in distilled water and degraded by H_2_O_2_ oxidative method. The influence of different conditions on the degradation of GFP was determined.

#### 2.1.1. Effect of Temperature

**Figure 1:**
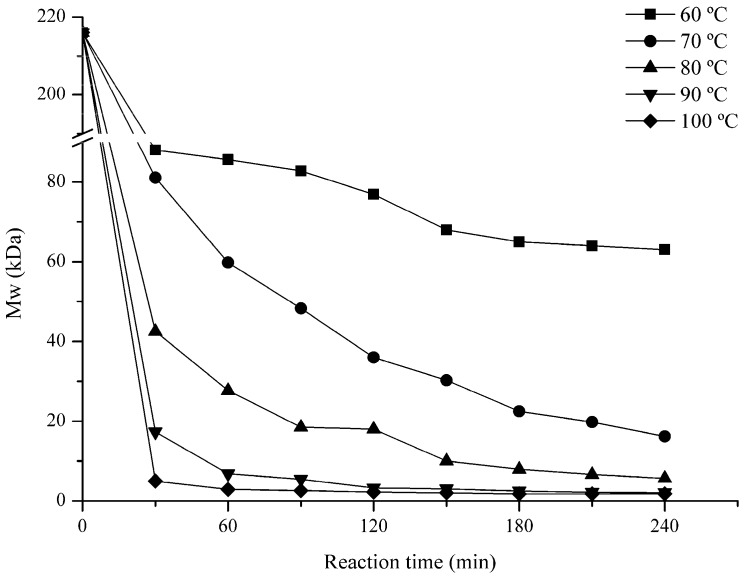
Effects of temperature on the molecular weight (Mw) of *Grateloupia filicinia* polysaccharide (GFP). The reaction was carried out in pH 1 and 0.15% H_2_O_2_ under different temperature conditions.

In order to investigate the effect of temperature on the Mw of GFP, 60 °C, 70 °C, 80 °C, 90 °C, and 100 °C were selected. As shown in Figure 1, the Mw of the products was high at a comparatively low temperature (such as 60 °C and 70 °C). At 120 min, the Mws changed to 76.9 kDa, 36 kDa and 18 kDa for 60 °C, 70 °C, and 80 °C, respectively. On the contrary, the Mws could violently decrease to 3.2 kDa and 2.2 kDa in 60 min when temperatures were 90 °C and 100 °C and the curves were tend to stay after 120 min. At last, the Mws of the polysaccharides for 60 °C, 70 °C, 80 °C, 90 °C, and 100 °C groups were 63 kDa, 16 kDa, 5.7 kDa, 2.0 kDa, and 1.8 kDa, respectively. The results showed that high temperature was more favorable for degradation and feasible to generate lower Mws polysaccharides.

#### 2.1.2. Effect of H_2_O_2_ Concentration

**Figure 2:**
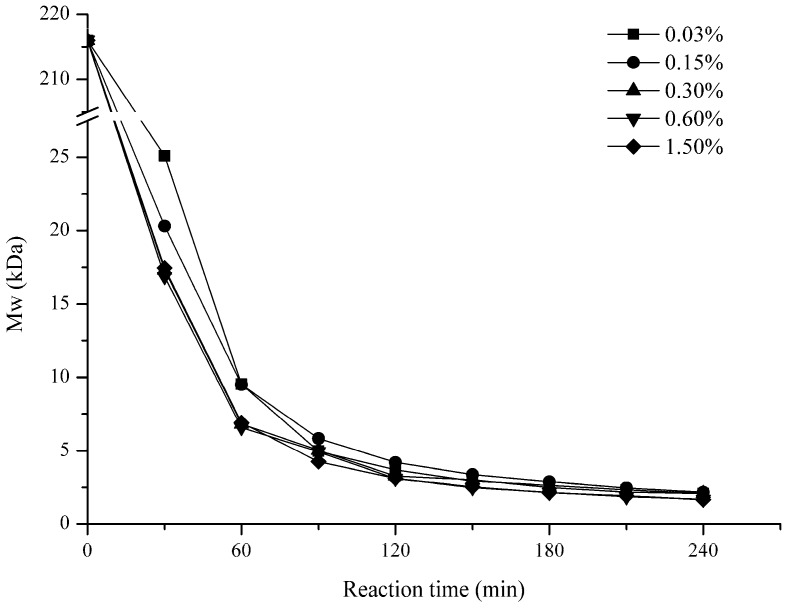
Effect of H_2_O_2_ concentration on the Mw of GFP. GFP was degraded at pH 1 and 90 °C under different H_2_O_2_ concentration conditions.

Figure 2 shows the effect of H_2_O_2_ concentration (0.03%, 0.15%, 0.30%, 0.6%, and 1.5%) on the Mw of the polysaccharides. In the first 60 min, Mw of 0.03% and 0.15% concentrations degradation products were similar and was bigger than the other groups, it decreased to about 9.5 kDa after 60 min. By contrast, the reaction with 0.3%, 0.6%, and 1.5% concentration groups presented no differences and were faster; the Mw of the products were all about 6.7 kDa after 60 min. However, after 90 min, the degradation results were similar for all of the groups. The curves of all H_2_O_2_ concentrations were closer, and the Mw of the final low molecular weight (LMw) polysaccharides were all around 2 kDa.

#### 2.1.3. Effect of pH

**Figure 3:**
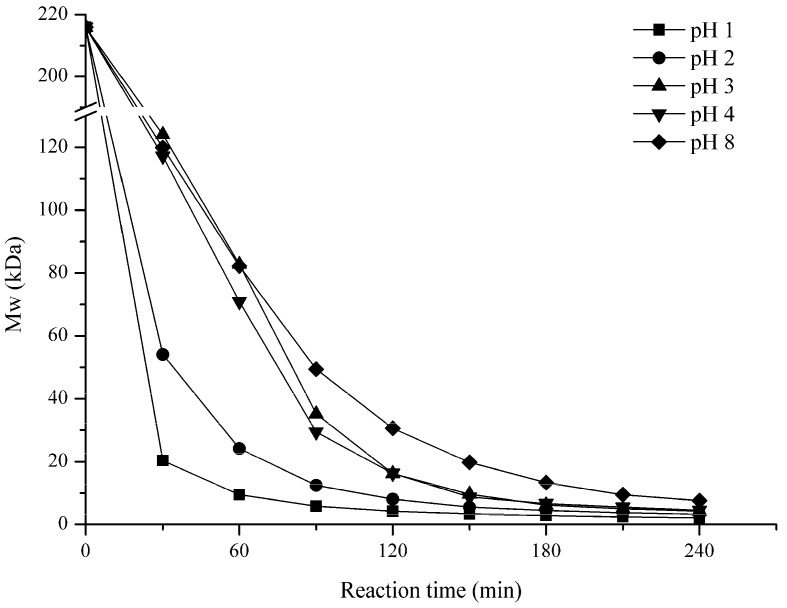
Effects of pH on the Mw of GFP. GFP was degraded in 0.3% H_2_O_2_ at 90 °C under different pH conditions.

Figure 3 illustrates the pH impact on the degradation of GFP. (pH 1, 2, 3, 4, and 8) were tested. As shown in Figure 3, the pH had a significant influence on GFP degradation. In 30 min, the Mw were remarkably reduced to 20 kDa and 54 kDa when under the lowest pH 1 and 2, respectively; but when pH was increased to 3, 4, or 8, the Mw of GFP were approximately 120 kDa at 30 min. After 120 min, the Mw of GFP changed slightly to a final Mw of 2.1 kDa and 3.2 kDa for pH 1 and 2, respectively. However, when the pH increased to 3, 4, or 8, the Mw decreased drastically to 4.2 kDa, 4.5 kDa and 7.6 kDa at 240 min, respectively. This suggested that the higher pH (such as 3, 4, and 8) could also degrade GFP, but a lower pH would accelerate the reaction to a shorter time.

### 2.2. Preparation and Chemical Characterization of LGFP

#### 2.2.1. Preparation of LGFP

Depending on the influence of different conditions on the degradation of GFP, a series of conditions were screened to prepare four kinds of LGFP solutions. After being neutralized to pH 7, the solution was dialyzed against tap water for 24 h and was then changed to distilled water for 24 h. The dialysis tube for LGFP-1 to LGFP-4 were 10 kDa, 3.5 kDa, 1 kDa, and 0.5 kDa Mw cut off, respectively. After dialysis, the solutions were filtered by common filter paper, freeze dried, and then the yield was calculated. The specific preparation conditions and yield are shown in Table 1.

#### 2.2.2. Chemical Characterization

**Figure 4:**
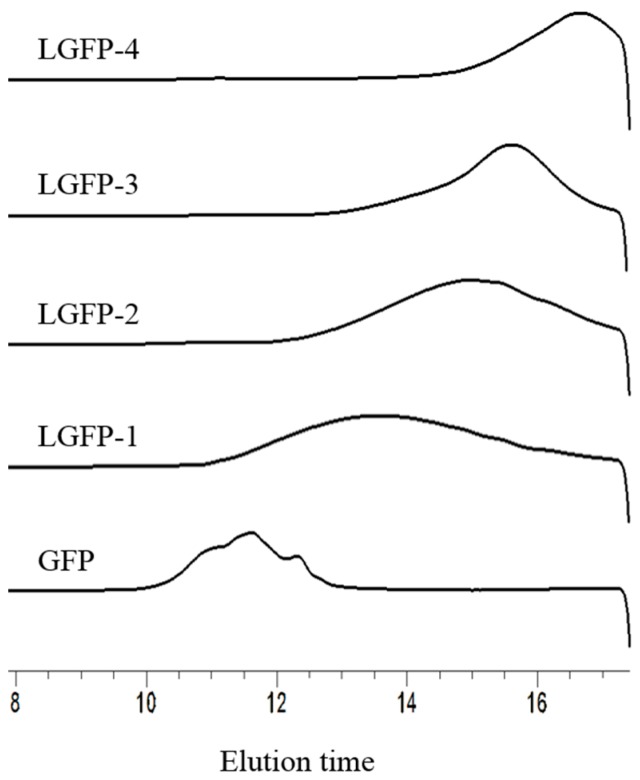
HPLC profiles of the GFP and LGFPs.

According to the conditions exhibited in Table 1, four LGFPs with different Mw were prepared. The HPLC profiles and chemical composition of the initial GFP and the LGFPs are given in Figure 4 and Table 2. The HPLC standard curve equation was *y* = −2.4616*x* + 24.507 with the Log Mw as the abscissa and elution time as the ordinate. The *R* square was 0.991. With the decrease of Mw of the polysaccharides, the total sugar content has decreased as well, but the sulfate content decreased slightly. The protein content of LGFPs also reduced. Results of monosaccharide composition showed that all samples were mainly composed of galactose.

**Figure 5:**
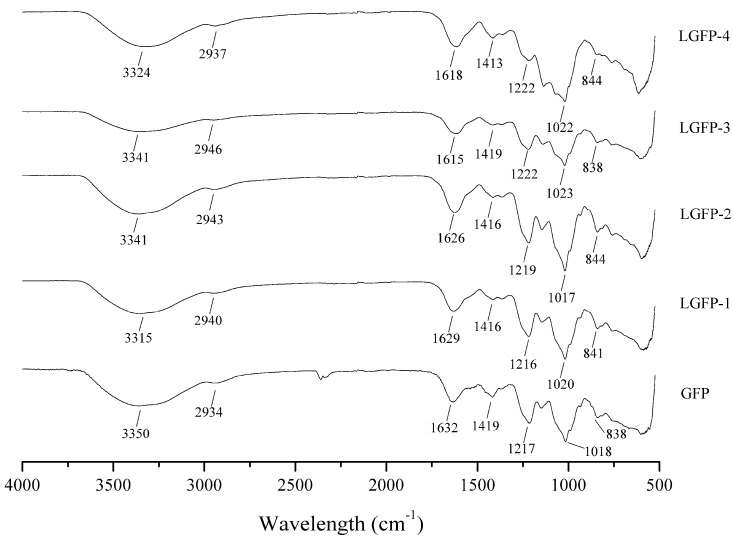
Fourier transform infrared (FT-IR) spectra of GFP and LGFPs in regions from 4000 to 500 cm^−1^.

In order to further characterize the chemical structure of the GFP and LGFPs, the respective FT-IR spectra were examined (Figure 5). Based on previous reports [17,23,24], the O–H stretching vibration appeared at 3300 cm^−1^, and C–H stretching vibration appeared at 2940 cm^−1^. The absorption peaks at 1620 cm^−1^ and 1420 cm^−1^ represents the asymmetric and symmetric stretching vibration of C=O, respectively. The absorption peaks at 1220 cm^−1^ indicates S=O stretching vibration and that at 1020 cm^−1^ correspond to C–O–H deformation vibration. Feature absorption at 840 cm^−1^ reflects C–O–S symmetry stretching vibration. 

### 2.3. Cytotoxic Activity

The specific results of MTT (3-(4,5-dimethyl-2-thiazolyl)-2,5-diphenyl-2-H-tetrazolium bromide) assay used to detect the cytotoxicity of GFP and LGFPs on DF-1 cells were shown in Table 3. It was considered that the polysaccharides do not have cytotoxic activity to DF-1 cells as the relative survival is over 85% [25,26]. These results suggested that 2 mg/mL was still safe for all samples.

### 2.4. Anti-ALV-J Activity In Vitro

#### 2.4.1. ALV-Specific Antigen Detection

**Figure 6:**
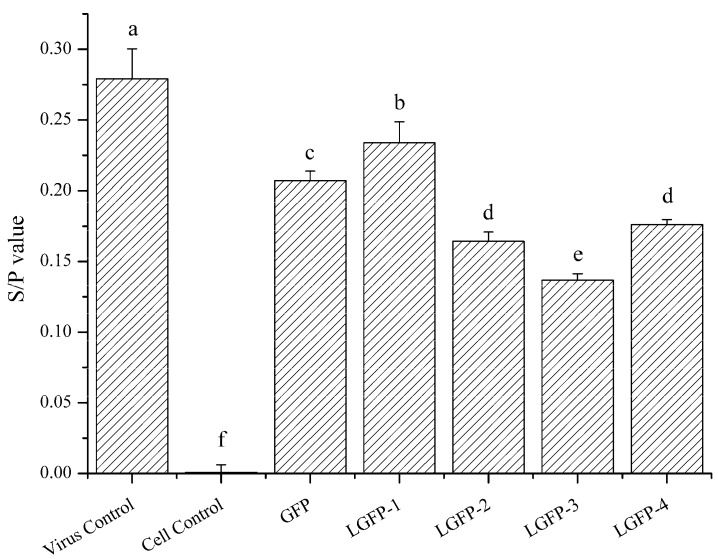
Expression of ALV-J p27 antigen. Antiviral activity of GFP and LGFPs were determined by ALV p27 antigen test kit. Results are recognized as positive when *S*/*P* value is greater than 0.2. Data are shown as the Mean + SD. Values with different letters in the same column (a–f) are significantly different (*p* < 0.05) from each other.

ELISA method was applied to preliminary verify the anti-ALV-J activity of polysaccharides preliminary. *S*/*P* value represented the relative expression of p27 antigen and was calculated by the following equation: *S*/*P* = (Sample mean − Negative control mean)/(Positive control mean − Negative control mean). As presented in Figure 6, under the concentration of 2 mg/mL, the *S*/*P* value of all of the tested groups were lower than the virus control, which means that the ALV p27 antigen of polysaccharides treated groups were dramatically lower than virus control. The effect of LGFP-2, LGFP-3, and LGFP-4 was better than that of GFP and LGFP-1, which possess higher Mw. Among them, LGFP-3 showed the best anti-ALV-J activity with a *S*/*P* value of 0.13, thus, LGFP-3 was cautiously chosen for further study.

#### 2.4.2. Action Phase of Polysaccharides

**Figure 7:**
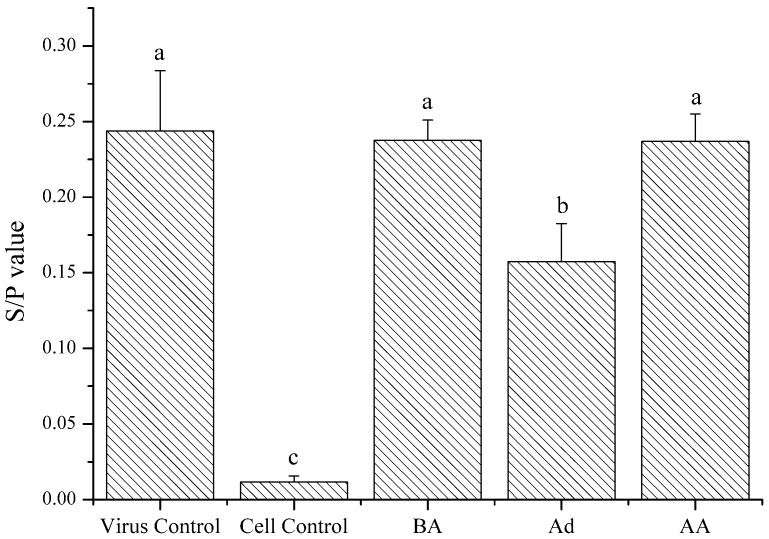
Expression of p27 after different modes of administration: LGFP-3 treated DF-1 cells before adsorption (BA); LGFP-3 treated virus at the adsorption phase (Ad); and, LGFP-3 treated DF-1 cells after adsorption (AA). The final concentrations of LGFP-3 were all 1 mg/mL in these three administration. DF-1 cells with and without inoculation were used as the virus and cell control, respectively. Data are shown as the Mean + SD. Values with different letters in the same column (a–c) are significantly different (*p* < 0.05) from each other.

Different plant polysaccharides exerted various antiviral characteristics [1,27,28]. In order to explore the action phase of the samples, LGFP-3 was assayed following different conditions, as indicated in Section 4.7.2. All of the treatments were applied to cells and virus in vitro, and the antiviral activity was detected by ELISA. The results showed in Figure 7 illustrated that the *S*/*P* value of Ad group was 0.15, significantly lower than the virus control that was 0.24, and also lower than that of BA and AA. This means that the GFP-3 might inhibit virus adsorption onto the DF-1 cells. The p27 expression of BA and AA (both 0.23) groups was lower than the virus control but the difference was not significantly (*p* > 0.05), which indicated that the treatment with polysaccharides before or after virus inoculation did not significantly decrease the virus infection.

#### 2.4.3. Gene Relative Expression of ALV-J

**Figure 8:**
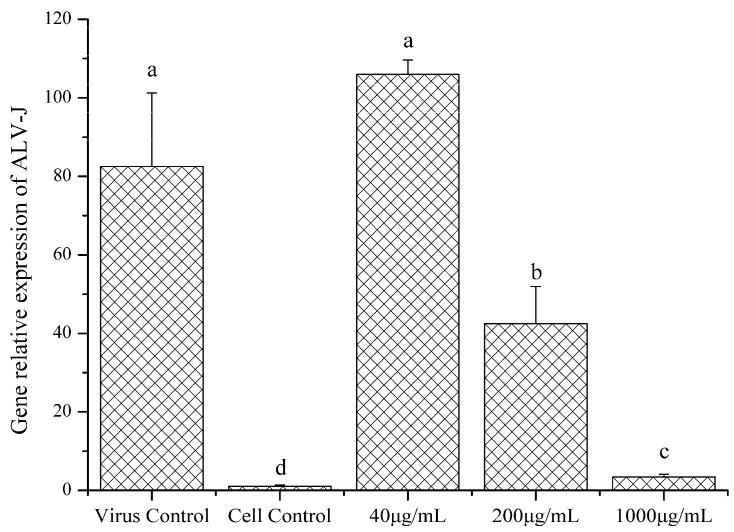
Gene relative expression of ALV-J measured with real-time PCR. DF-1 cells with or without inoculation were used as the virus and cell control, respectively. Data are shown as the Mean + SD. Values with different letters in the same column (a–d) are significantly different (*p* < 0.05) from each other.

Real time PCR was performed to evaluate the ALV-J gene expression with or without LGFP-3 treatment. The results were presented in Figure 8. The expression of ALV-J gene decreased significantly after treatment with LGFP-3 (1000 and 200 μg/mL), and the suppression was dose-dependent. Treatment with 1000 μg/mL LGFP-3 caused the strongest inhibition against ALV-J adsorption; the relative gene expression of 200 μg/mL in the experiment group was 42.52, weaker than 1000 μg/mL group but significantly lower than virus control. While handling with 40 μg/mL LGFP-3, the gene expression was higher than the other two concentrations and even higher than the virus control. This suggested that under a low concentration, the LGFP might promote ALV-J adsorption.

#### 2.4.4. Western-Blot and Indirect Immunofluorescence Assay (IFA) Analysis

**Figure 9:**
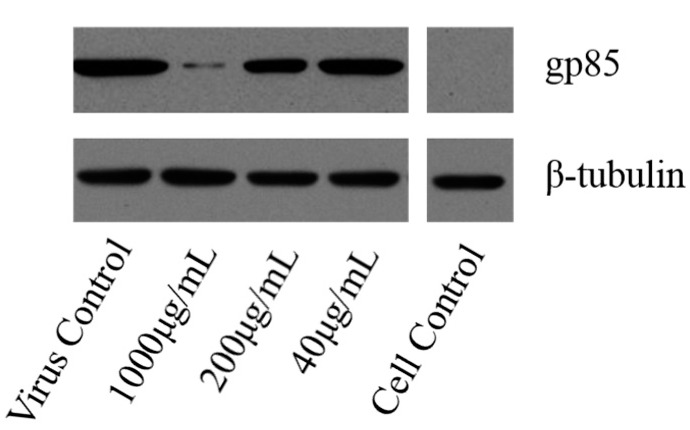
Expression of ALV-J gp85 protein evaluated by western-blot. DF-1 cells with or without inoculation were used as the virus and cell control, respectively.

**Figure 10:**
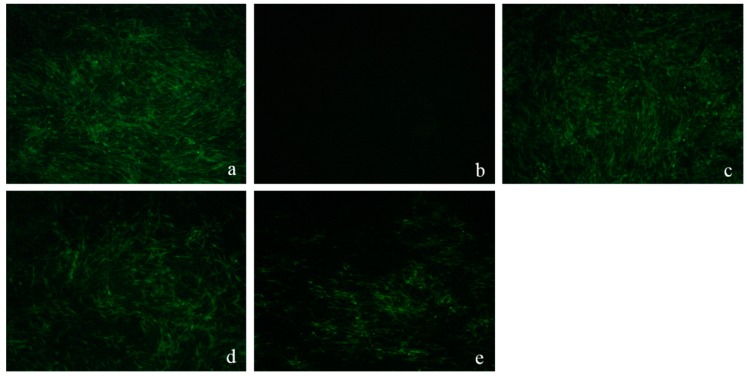
Expression of ALV-J gp85 protein evaluated by IFA. (**a**): virus control; (**b**): cell control; (**c**): ALV-J treated with 40 μg/mL LGFP-3; (**d**): ALV-J treated with 200 μg/mL LGFP-3; and, (**e**): ALV-J treated with 1000 μg/mL LGFP-3.

To further investigate the variation of ALV-J when treated with LGFP-3 as compared to cell and virus control, western-blot and IFA were also used; the results were shown in Figure 9 and Figure 10, respectively. As it happened with the results of real time PCR, in western-blot analysis, expression of ALV-J gp85 protein was almost the same as virus control when treated with 40 μg/mL LGFP-3. However, when the concentration was 1000 μg/mL, the gp85 protein expression significantly decreased and gained a much greater effect when compared with 200 μg/mL and 40 μg/mL LGFP-3. These results also showed that the antiviral effects were dose dependent.

A more direct observation of DF-1 cells infected by ALV-J could be obtained from IFA. Results showed that five days after inoculation, the green fluorescence signal intensity of 40 μg/mL group was the strongest and similar to that of the virus control. The majority of the DF-1 cells were also infected with ALV-J in the 200 μg/mL group but less in number than the virus control group. Cells that were treated with 1000 μg/mL LGFP-3 had the lowest virus expression. The antiviral effects increased following by the increase of LGFP-3 concentration, a reaction to that real time PCR and Western blot.

## 3. Discussion

The GFP was recognized for its various biological activities [14,29,30]. However, researches focusing on the degradation of GFP and antiviral activity of LGFP are still scarce. Therefore, it is necessary and significant to explore the influence of degradation conditions on the Mw of GFP and the antiviral activity of LGFP. For all of the degradation methods, H_2_O_2_ oxidative degradation was characterized for its mild reaction and low cost; besides, the unreacted excess H_2_O_2_ is easy to remove. Because of the advantages mentioned above, H_2_O_2_ oxidative degradation has been widely used in degrading polysaccharides such as chitosan, starch, and cellulose [31,32,33]. Further, the pH, H_2_O_2_ concentration and temperature were considered to have a great impact on the degradation rate [17,34]. 

In this study, the GFP degradation rate was shown to be positively related to temperature, H_2_O_2_ concentration, and negatively to pH in general. Probably, during the degradation process, H_2_O_2_ could generate free radicals such as HOO^−^, ^•^O_2_, and HO^•^, which could remove a H atom bonded to contributing to the fracture of the glycosidic linkages [35]. Our results showed that the degradation for 0.03% and 0.15% H_2_O_2_ were slower than that of 0.3% in the first 90 min. This might suggest that a higher H_2_O_2_ concentration meant that more free radicals are produced in solution, therefore accelerating the degradation. When considering that the degradation results for 0.6% and 1.5% H_2_O_2_ were same as the 0.3% from the beginning to the end, so, concentration at 0.3% was enough for GFP degradation. However, after 90 min, the 0.03% and 0.15% groups were gradually approached to the 0.3% group, and the final results were basically the same for the three groups. This suggests that a high H_2_O_2_ concentration might speed up the degradation rate, but it dose not influence the final results. Another parameter that affects the degradation of GFP might be pH. For example, Iqbal et al. degraded dextran at 80 °C for 2 h, and verified that the Mw of the product was 270 kDa with pH of 1.8, while the Mw decreased to only 77 kDa for pH 1.4 [36]. In our study, compared with pH 3, 4, and 8, pH 1 and 2 could significantly accelerate the degradation probably because pH could also affect the generation rate of free radicals in the solution. Therefore, in an acidic environment provided by a low pH, polysaccharides could be degraded more easily. Another important factor in GFP degradation is temperature. As shown in Figure 3, 10 kDa products could be obtained within 60 min at 90 °C and 100 °C, but at 60 °C and 70 °C, the Mw of the products were 63 kDa and 16 kDa only after 240 min. Hence, the Mw of the degradation products decreased significantly with the increase of temperature. There, we adopted 90 °C to prepare LGFPs (Table 1).

Based on the degradation results, several LGFPs were prepared and the chemical composition of GFP and LGFPs were also characterized. According to the results, we found that the protein content were reduced with the decrease of Mw in general, which indicated that the degradation was a benefit for protein removal. Along with the decrease of Mw, and the total sugar content also decreased, it might because of the comprehensive effect, which is caused by the temperature, H^+^, and free radicals in the degradation process. The monosaccharide composition results showed that all of the samples were mainly composed of galactose, the other monosaccharide content was extremely low. The pattern mentioned in FT-IR indicated that GFP and LGFPs were all sulfated polysaccharides and the degradation process did not change the chemical structure of GFP.

ALV-J has gradually spread in China and caused great losses to poultry breeding industry [37,38,39]. When compared with other ALVs, high recombination of ALV-J and selection pressure lead to the faster variation rate [40,41], which caused great difficulties in preventing and treating ALV-J outbreaks. 

Polysaccharides from the algae were found to exert inhibition to many viruses [42,43,44]. To verify the suppression of GFP and LGFPs to ALV-J, the ALV p27 antigen was detected in the presence or absence of the polysaccharides. P27 antigen is a common protein in ALVs, but it is often used to determine the ALV-J infection levels in the laboratory when the pathogen is ensured. Results in the study showed that all of the samples could significantly inhibit the expression of p27 antigen, and LGFP-2, 3, and 4 were better than GFP and LGFP-1. Among them, LGFP-3 with a Mw of 8.7 kDa exerted the best inhibition effect. 

In order to determine the function time-point of polysaccharides on ALV-J, LGFP-3 was chosen for further experiments. Figure 5 demonstrated that p27 antigen levels reduced only when the virus and LGFP-3 were administered simultaneously. Further, the BA and AA treatments were similar to the virus control. Therefore, the polysaccharides might mainly inhibit ALV-J adsorption on host cells. This was also observed in some other reports. Bouhlal et al., for example, found that polysaccharides extracted from *Sphaerococcus coronopifolius* and *Boergeseniella thuyoides* inhibited HSV-1 adsorption onto host cells [28], and Bourgougnon et al. suggested that the polysaccharides from *Schizymenia dubyi* could suppress HIV and the activity was mainly attributed to the inhibition of virus to cell attachment [45]. While Wang et al. found that fucoidan from *Kjellmaniella crassifolia* could inhibit the influenza A virus (IAV) treated with fucoidan before or after inoculation [27]. It seems that the action phase of fucoidan was not the same as LGFP. This is probably because the monosaccharide composition and the chemical structure of fucoidan, and also the viruses were different from this study. In addition, many researches showed that the sulfate content played a very important role in polysaccharides activities [46,47,48,49]. However, in this research, although the inhibition effect of LGFP-3 was the best, the sulfate content of LGFP-3 was not the highest. Perhaps, the inhibition effect of GFP and LGFPs to ALV-J was not only related to the sulfate content but also to the Mw of the polysaccharides or the sulfate pattern of distribution. According to the results, we may deduce that LGFP with a Mw of about 8.7 kDa might bind to some specific receptors on the cells with a more suitable structure and ionic interactions between polysaccharides and cells, or it could fully bind to ALV-J particles and block the specific recognition between virus and cells, thereby preventing the virus from adsorption. In order to further prove the LGFP-3 anti-ALV-J effect, virus genes, and specific protein expression were tested by real-time PCR, Western-blot and IFA. A significant suppression of ALV-J gene and gp85 protein expression were detected. Based on the real-time PCR results, gene expression of virus control were 24-times higher than that of 1000 μg/mL LGFP-3 group, thus the virus replication was significantly decreased. From the western-blot and IFA results, it can be observed that the virus protein expression and the number of ALV-J decreased significantly in the groups under 1000 and 200 μg/mL treatment. Thus the quantity of viral protein synthesis was also reduced. All of the above results showed that LGFP-3 could inhibit the ALV-J adsorption and reduce the infection probability of virus. Similar results were obtained by Yu et al., who discovered that the polysaccahrides from Taishan *Pinus massoniana* pollen could directly coat to the virus envelope protein, which would bind to the cell surface receptor, thereby affecting the virus adsorption [1]. Because LGFP-3 had no impact on ALV-J when applied before adsorption (BA), we speculated that our samples could not bind to the cell membrane, and the possible way was to interact with the virus envelope protein just like the polysaccahrides from Taishan *Pinus massoniana* pollen. But, considering the complex configuration of our samples, the action targets might be more and the specific mechanism needs to be elucidated in the future.

## 4. Materials and Methods

### 4.1. Seaweeds Samples and Reagents

*G. filicina* was collected from the number two bathing beach of Qingdao, China in January 2016. The seaweeds were washed with distilled water to remove salt, sediment, and other impurities, then dried at 50 °C in an oven to constant weight and stored at room temperature. All of the reagents used were of analytical grade.

### 4.2. Extraction of GFP

Traditional hot water extraction and alcohol precipitation method [26] was used with some modifications. Briefly, the dried algae were mixed with 60-fold volume of distilled water and maintained at 100 °C for 4 h. After cooling down, the polysaccharide solution was filtered and condensed to 1/2 volume by a rotavapor. Then, the supernatant was dialyzed at tap water for 24 h and then changed to distilled water for 24 h, respectively, using dialysis tube with a 3.5 kDa Mw cut off. The liquid was concentrated to 1/4 volume and after that 3-fold volume anhydrous ethanol was added to precipitate polysaccharides. The mixture was placed over-night at 4 °C and then centrifuged. The precipitate was freeze-dried and the yield (%) of the polysaccharide was calculated.

### 4.3. Influence of H_2_O_2_ Oxidation on GFP Degradation and Preparation of LGFP

A 0.8 g mass of GFP was dissolved in 40 mL distilled water, added with H_2_O_2_ solution, and adjusted pH by hydrochloric acid or sodium hydroxide, and then heated in a water bath with stirring. Every 30 min, 0.5 mL samples were removed from the reaction solution. After being filtrated with a 0.22 μm polyethersulfone filter (Tianjin Jinteng Experiment Equipment Co., Ltd, Tianjin, China), the Mw of the samples was determined by high performance gel permeation chromatography (HPGPC) to investigate the effect of pH (1, 2, 3, 4, and 8), final concentration of H_2_O_2_ (0.03%, 0.15%, 0.3%, 0.6%, and 1.5%) in reaction solution and temperature (60 °C, 70 °C, 80 °C, 90 °C, and 100 °C) on the GFP degradation. Afterwards, according to the above results, LGFPs were prepared under different conditions.

### 4.4. Chemical Characterization

The Mw of GFP and LGFPs was measured by HPGPC method with a TSK gel G3000PWxl column, and using 0.1 mol/L Na_2_SO_4_ as the mobile phase on Agilent 1260 HPLC systerm equipped with a refractive index detector. The column temperature was 35 °C and flow rate was 0.5 mL/min. Dextran standards (Mw 1000, 5000, 12,000, 50,000, 80,000, 270,000, and 670,000, Sigma, Mendota Heights, MN, USA) were used to calibrate the column.

Total sugars were analyzed by phenol-sulphuric acid method [50] using galactose as the standard. Bradford’s method [51] was used to detect the protein content and using bovine serum albumin (BSA) as the standard. Sulfated content was determined by barium chloride gelatin method [52]. The Fourier transform infrared (FT-IR) spectra of the samples were recorded by Nicolet-360 FT-IR spectrometer (Thermo Fisher, Waltham, MA, USA) in KBr disks using a scan range from 500 to 4000 cm^−1^.

The monosaccharide composition (molar ratio) was analyzed using 1-phenyl-3-methyl-5-pyrazolone (PMP) pre-column derivation HPLC [53]. Briefly, 10 mg samples were put into the ampoule and 1 mL distilled water was added then the mixture was hydrolyzed in 4 mol/L trifluoroacetic acid for 4 h at 110 °C in an oven followed by neutralization with sodium hydroxide to pH 5–6. Later, the pre-column derivatives were carried out with PMP and separated by HPLC using a YMC Pack ODS AQ column. The mannose, rhamnose, fucose, galactose, xylose, glucose, and glucuronic acid standards were obtained from Sigma Aldrich (St. Louis, MO, USA).

### 4.5. Cell Lines, Cell Culture, Virus and Antibodies

A DF-1 cell line, a NX0101 strain of ALV-J, and the ALV-J gp85-specific monoclonal antibody were kindly gifted by Prof. Cheng, Shandong Agricultural University. The DF-1 cells were cultured in Dulbecco’s modified Eagle’s medium (DMEM) provided with 100 units/mL of penicillin and 100 μg/mL of streptomycin, and supplemented with 10% (*v*/*v*) or 1% (*v*/*v*) fetal bovine serum (FBS) as the growth medium (GM) or maintenance medium (MM), respectively. The ALV-J titer was tested using DF-1 monolayers and expressed by tissue culture infectious dose 50 (TCID_50_) using the Reed-Muench formula. 100 TCID_50_ of ALV-J was used in subsequent experiments.

### 4.6. Determination of Cytotoxic Activity

MTT assay was used to evaluate the cytotoxic activity of GFP and LGFP on DF-1 cells. DF-1 cell monolayers grown in 96-well plate were supplied with the 100 μL MM as the cell control or 100 μL polysaccharides solution that were dissolved in MM (the maximum concentration was 2 mg/mL and diluted in 2-fold steps to 0.03125 mg/mL). All of the treatments were performed in triplicate. After 24 h, the liquid was removed; 20 μL MTT was added and the cells were incubated for another 4 h. Then, the MTT was discarded and 100 μL DMSO was added. Finally, the enzyme-linked immunosorbent assay reader (iMark^TM^ BIO-RAD, Hercules, CA, USA) was used to determine the absorbance of each well at 490 nm. The cytotoxic activity was represented by the relative survival rate of DF-1 cells and calculated by the formula: survival rate (%) = (*As*/*Ac*) × 100%, where *Ac* and *As* were the absorbance of cell control (*Ac*) and experimental group (*As*), respectively. When the survival rates were higher than 85%, it was considered that the samples had no cytotoxicity on DF-1 cells.

### 4.7. Anti-ALV-J Activity In Vitro

#### 4.7.1. ALV Specific Antigen Detection

DF-1 cell monolayers grown in 96-well plate were infected with 100 TCID_50_ of ALV-J, meanwhile, different polysaccharides solutions at a final concentration of 2 mg/mL, which were dissolved in MM were added. After 2 h incubation at 37 °C, the cells were washed and covered with MM containing corresponding polysaccharides at a concentration of 2 mg/mL, subsequently, the cell control (without infection of ALV-J) and virus control (without exposure to polysaccharides) were also set. After 24 h of incubation, the viral titers were measured using the ALV p27 antigen test kit (Beijing IDEXX-Yuanheng Laboratories, Co. Ltd., Beijing, China) following by the instructions of the manufacturer. The relative expressions of p27 antigen were calculated by the following equation: *S*/*P* = (Sample mean − Negative control mean)/(Positive control mean − Negative control mean).

#### 4.7.2. Detection of the Polysaccharides Action Phase

DF-1 cells grown in 96-well plate were infected with 100 TCID_50_ of ALV-J. Then, they were treated with MM containing polysaccharides at a final concentration of 1 mg/mL in different ways. According to the artificially divided viral infection phase, the specific operations were as follows:

Before Adsorption (BA): The DF-1 cells were covered with MM containing polysaccharides for 2 h before the inoculation. After that, the cells were washed and then incubation with ALV-J for 2 h. After inoculation, the cells were washed again and maintained with MM for 24 h.

Adsorption (Ad): The cells were treated with MM containing polysaccharides at the time they were infected with ALV-J. After incubation at 4 °C for 2 h, the supernatant was removed and replaced by simple MM.

After Adsorption (AA): The DF-1 cells were infected with ALV-J and incubated for 2 h, and then the cells were washed and recovered with polysaccharides dissolved in MM.

The cell control and virus control were also served. After 24 h incubation, all of the supernatants were collected and the viral titers were measured by ALV p27 antigen test kit.

#### 4.7.3. Viral Gene Relative Expression and Protein Expression

DF-1 cells grown in 12-well plate to monolayers in simple MM medium were infected with 100 TCID_50_ of ALV-J and treated with MM containing polysaccharides at a final concentration of 1000 μg/mL, 200 μg/mL, and 40 μg/mL. After 2 h adsorption at 37 °C, the supernatant was discarded and replaced by simple MM, then the cells were incubated at 37 °C with 5% CO_2_ for about 24 h.

Real-time PCR: The ALV-J gene replication levels in DF-1 cells were determined by real-time PCR. In brief, RNAprep Pure Cell/Bacteria Kit (TIANGEN BIOTECH Co. Ltd., Beijing, China) was used to extract the total RNA from the treated DF-1 cells following the manufacturer’s instructions. Then, PrimeScript^TM^ RT reagent Kit with gDNA Eraser (Takara BIO INC, Dalian, China) was applied for reverse transcription immediately. The cDNA produced by reverse transcription was used for real-time PCR as performed by SYBR^®^ *Premix ExTaq*^TM^ Kit (Takara BIO INC, Dalian, China). The forward primer (5′-GCGTGCGTGGTTATTATTTC-3′) and reverse primer (5′-AATGGTGAGGTCGCTGACTGT-3′) were used as the ALV-J primers, the forward primer for internal control GAPDH was (5′-GAACATCATCCCAAGCGTCCA-3′) and reverse primer (5′-CGGCAGGTCAGGTCAACAAC-3′). The amplification cycles were carried out as follows: 95 °C for 30 s; 95 °C for 5 s and 60 °C 34 s (34 cycles); 95 °C for 15 s and 60 °C for 60 s. The results were calculated by the formula 2^−ΔΔ*C*t^ [54,55].

The expression of ALV-J gp85 protein was detected by western blot and IFA.

Western-blot: After 24 h incubation, the cells were lysed and separated by SDS-PAGE (12% acrylamide gels). Proteins were blocked with Tris-buffered saline (TBS) buffer containing 5% (*w*/*v*) dry skimmed milk, and 0.1% (*v*/*v*) Tween 20 for 1 h at room temperature. Membranes were then incubated overnight at 4 °C with mouse ALV-J gp85-specific monoclonal antibody or β-tubulin antibody as control. The membranes were washed with TBS-Tween and then incubated with HRP-linked secondary antibody (1:10,000) for 1 h at room temperature. Membranes were washed and protein densities were detected using chemiluminesence detection reagents. 

IFA: After inoculation, the DF-1 cells were incubated at 37 °C with 5% CO_2_ for five days and then IFA was executed as follows. The cells were fixed with cold acetone and ethanol (3:2 for *v*/*v*) for about 10 min washed with PBS and the gp85-specific monoclonal antibody was added. The mixture was incubated overnight at 4 °C and washed thoroughly with PBS to eliminate the uncombined antibodies. Afterwards, the cells were treated with goat anti-mouse IgG-FITC at 37 °C for 1 h, washed with PBS for five times and then photographed on an inverted fluorescence microscope.

### 4.8. Statistical Analysis

Statistical analysis was performed using SPSS and the difference among groups was analyzed by one-way ANOVA.

## 5. Conclusions

In this study, the polysaccharides (Mw 216 kDa) extracted from *G. filicina* were degraded by H_2_O_2_ oxidative method. The degradation rate was positively related to temperature, H_2_O_2_ concentration, and negatively to high pH. Several LGFPs (Mw 40, 14, 8.7, and 2.7 kDa) were prepared under different conditions and their anti-ALV-J activity were also investigated. The results showed that initial GFP and LGFPs all possessed antiviral activity, and low Mw is better for the inhibition of ALV-J, LGFP-3 (Mw 8.7 kDa) had the best effect. The action phase of our samples was also determined. The results showed that LGFP-3 were mainly take effect when treated by Ad administration the time that ALV-J adsorbing on host cells. Further experiments indicated that after being treated with LGFP-3, the ALV-J gene and protein expression decreased significantly: it revealed that LGFP-3 inhibited virus adsorption onto DF-1 cells and contributed to the decrease of ALV-J expression. The results demonstrated that polysaccharides from *G. filicina* have great potential in developing as an anti-ALV-J drug. However, the antiviral activity of LGFPs in vivo and complicated mechanism in detail require further study.

## Figures and Tables

**Table 1:** Preparation conditions and yield of LGFPs.

Sample	Temperature (°C)	pH	H_2_O_2_ (%)	Time (min)	Yield (%)
LGFP-1	90	4	0.3	75	73.4
LGFP-2	90	4	0.3	135	46.28
LGFP-3	90	4	0.3	210	42.99
LGFP-4	90	4	0.3	240	40.38

**Table 2:** Chemical composition of polysaccharides (%*w*/*w* of dry weight).

Sample	Total Sugar (%)	Sulfate (%)	Protein (%)	Mw (kDa)	Monosaccharides Composition (Molar Ratio)						
					Man	Rha	Glc A	Glc	Gal	Xyl	Fuc
GFP	55.22 ± 0.96	21.52 ± 0.04	1.45 ± 0.08	216.7 ± 1.2	0.00	0.01	0.03	0.01	1	0.03	0.03
LGFP-1	49.74 ± 1.22	20.99 ± 0.28	0.96 ± 0.01	40.2 ± 0.3	0.01	0.01	0.03	0.01	1	0.03	0.02
LGFP-2	47.21 ± 1.32	16.86 ± 0.10	0.45 ± 0.02	14.0 ± 0.3	0.01	0.01	0.05	0.01	1	0.03	0.02
LGFP-3	44.73 ± 2.43	18.33 ± 0.43	0.18 ± 0.01	8.7 ± 0.2	0.01	0.01	0.05	0.01	1	0.03	0.01
LGFP-4	41.00 ± 0.25	17.01 ± 0.44	0.29 ± 0.00	2.7 ± 0.1	0.01	0.02	0.04	0.01	1	0.02	0.02

Man: mannose; Rha: rhamnose; Glc A: glucuronic acid; Glc: glucose; Gal: galactose; Xyl: xylose; Fuc: fucose.

**Table 3:** Relative Survival Rate of DF-1 cells.

Concentration (mg/mL)	2	1	0.5	0.25	0.125	0.0625	0.03125
GFP	0.95 ± 0.02	1.00 ± 0.06	1.03 ± 0.07	1.08 ± 0.01	1.07 ± 0.04	1.05 ± 0.09	1.02 ± 0.06
LGFP-1	0.96 ± 0.02	1.00 ± 0.05	1.05 ± 0.04	1.05 ± 0.08	1.01 ± 0.04	0.99 ± 0.04	1.00 ± 0.04
LGFP-2	0.9 4± 0.05	1.06 ± 0.04	1.01 ± 0.02	1.05 ± 0.04	1.05 ± 0.05	1.01 ± 0.04	1.03 ± 0.07
LGFP-3	0.9 5± 0.02	1.02 ± 0.05	1.04 ± 0.10	0.99 ± 0.03	1.03 ± 0.03	0.99 ± 0.05	1.03 ± 0.04
LGFP-4	0.96 ± 0.10	0.98 ± 0.03	1.03 ± 0.06	1.01 ± 0.05	1.01 ± 0.03	1.04 ± 0.07	1.09 ± 0.03

The safe concentration of GFP and LGFPs was tested using MTT assay. All treatments were performed in triplicate. The relative survival rate reflected the cytotoxity of GFP and LGFPs.
